# The use of a portfolio in postgraduate medical education – reflect, assess and account, one for each or all in one?

**DOI:** 10.3205/zma001134

**Published:** 2017-11-15

**Authors:** Sylvia Heeneman, Erik W. Driessen

**Affiliations:** 1 Maastricht University/MUMC, Department of Pathology, HX Maastricht, The Netherlands; 2 Maastricht University, School of Health Professions Education, Faculty of Health, Medicine and Life Sciences, Maastricht, The Netherlands; 3 Maastricht University/MUMC, Department of Educational Development and Research, HX Maastricht, The Netherlands

**Keywords:** postgraduate education, feedback, portfolio, reflection, quality insurance

## Abstract

Competency-based education has become central to the training and assessment of post-graduate medical trainees or residents [1]. In competency-based education, there is a strong focus on outcomes and professional performance. Typically, holistic tasks are used to train, practice and assess the defined outcomes or competencies. In residency training, these tasks are part of the day-to-day clinical practice. The performance of residents in the workplace needs to be captured and stored. A portfolio has been used as an instrument for storage and collection of workplace-based assessment and feedback in various countries, like the Netherlands and the United States. The collection of information in a portfolio can serve or be used for a variety of purposes. These are:

1. The collection of work samples, assessment, feedback and evaluations in a portfolio enables the learner to look back, analyze and reflect.
2. The content is used for assessment or making decisions about progress. And
3. the portfolio is used as an instrument for quality assurance processes.

In post-graduate medical education, these purposes can be combined but this is not always reported transparently. In this paper, we will discuss the different perspectives, how a portfolio can serve these three purposes and what are opportunities and challenges of combining multiple purposes.

## Zusammenfassung

Die kompetenzbasierte Ausbildung ist zum zentralen Element in der Weiterbildung und Beurteilung von postgraduierten medizinischen Auszubildenden oder Assistenzärzten geworden [1]. In kompetenzbasierter Ausbildung gibt es eine klare Fokussierung auf Ergebnisse und professionelle Leistungen. Typischerweise werden holistische Aufgaben verwendet, um definierte Ergebnisse oder Kompetenzen zu üben, zu praktizieren und zu bewerten. Während der Facharztausbildung sind diese Aufgaben Teil des klinischen Alltags. Die Leistung der Assistenzärzte am Arbeitsplatz muss erfasst und dokumentiert werden. Ein Portfolio ist als Instrument für die Speicherung und Sammlung von Beurteilung und Feedback am Arbeitsplatz in verschiedenen Ländern, wie den Niederlanden und den Vereinigten Staaten, verwendet worden. Die Sammlung von Informationen in einem Portfolio kann verschiedenen Zwecken dienen oder für verschiedene Zwecke verwendet werden. Diese sind:

1. Die Sammlung von Arbeitsproben, Beurteilungen, Feedback und Evaluationen in einem Portfolio ermöglicht dem Lernenden zurückzublicken, zu analysieren und zu reflektieren.
2. Der Inhalt dient der Bewertung oder den Entscheidungen über den Fortschritt. Und
3. das Portfolio wird als Instrument für Qualitätssicherungsprozesse verwendet.

Innerhalb der postgraduierten medizinischen Weiterbildung können diese Zwecke kombiniert werden, aber dies wird nicht immer transparent berichtet. In diesem Artikel werden die verschiedenen Perspektiven erörtert, wie ein Portfolio diese drei Zwecke erfüllen kann und was die Möglichkeiten und Herausforderungen sind, um verschiedene Nutzungen zu kombinieren.

## Portfolios in post-graduate medical education

Portfolios are instruments to collect and assess evidence of the learners’ progression in tasks or competencies. The content can be prescribed or is to the choice of the learner. The performance and feedback information is used by the learner to show progress, look back and reflect on feedback, and formulate plans/ learning objectives for improving performance or competence [2], [3]. In the post-graduate setting, Smith and Tillema [4] made a differentiation in types of portfolios based on

1. the purpose of the portfolio, being either selection or developmentally orientated, and
2. the setting of use, being either mandated or self-directed.

This led to 4 types:

1. The dossier portfolio, containing mandated records of achievement, with specified levels of performance, used for selection or promotion.
2. The training portfolio, containing a mandated collection of acquired skills and competencies, in a fixed format, with some reflective comments on selected evidence.
3. The reflective portfolio containing a purposeful collection of evidence for personal growth and development, used for promotion or selection. And
4. the personal development portfolio, containing reflective and personal evaluations of progress in time, enabling discussion and valuing of these activities.

In practice, there may be more of a continuum. A portfolio in post-graduate education is often mandatory, containing prescribed information such as 360 degree feedback rounds, evaluations of clinical performance etc., and it is used for assessment. Therefore, it would be best typified as a training portfolio. However, portfolios in postgraduate education can also have a stronger developmental aspect, with more reflective accounts of personal and professional growth, used for a dialogue with a supervisor or mentor. This diversity in purposes is further discussed in the paragraphs below.

## The portfolio to reflect and learn

There are many studies on the effect of reflection in a portfolio on learning in the undergraduate setting. Through reflection, the learner can scrutinize the personal performance, analyse and come to terms with what went well and what went wrong and set out learning objectives for strategies to improve. This supports the learner taking responsibility for his or her learning [5]. Reflection was shown to have a small, positive impact on performance (case-solving capacity) [6]. In addition, a study by Sobral [7] showed that reflection led to progression in learning and was (weakly) correlated to improved academic performance. 

In postgraduate education, the learning is done at the workplace. There is some evidence that reflection on experience in the clinical workplace, as in the undergraduate setting, led to a deeper approach to learning, allowing integration of what was learned with existing knowledge and skills [8]. In addition, reflection improved the diagnostic reasoning on complex and unusual patients or cases [9].

The implementation of a portfolio can be used to facilitate or encourage reflection just by the fact that information is collected and reviewed by the learner. However, the perception of residents whether a portfolio should be a tool for reflection varied, ranging from a positive 72% of surgical residents [10] to 42% of General Practitioner trainees [11]. The lack of willingness to reflect in a portfolio could be caused by an uncertainty or lack of confidence on how this information is used [12], especially if the reflective activities are also assessed (see next paragraph). Another finding was that learners considered themselves already as reflective practitioners and the obligation to reflect in the portfolio actually hindered their own approach to professional learning [13]. In addition, time is an important factor. In workplace-based education, workload is high and the collection of workplace-based feedback and evaluation for the portfolio and having to reflect can be a burden [14], [15].

Lack of acceptance is an important issue. Portfolios need a supportive structure and cannot be implemented as an independent device. Research has shown that portfolios work if certain conditions have been fulfilled: mentoring, a structure that allows some flexibility for the learner (agency), an environment that supports learning and providing a meaningful learning experience for the learner [16]. For reflective activities in the portfolio, these conditions are very similar. Very often portfolios in post-graduate training have an electronic format, and this can be used to support or facilitate reflection, e.g. by the use of electronic devices [17], [18], [19]. Keim et al. [20] studied whether the introduction of a portfolio as such increased reflective activities and indeed showed more self-reflective entries in trainees with a portfolio, compared to a control group with no portfolio. The introduction of a smartphone App as support for reflection also increased reflective activities [19]. So, when conditions are met, this seems to result in better acceptance. It will however, remain a challenge to ensure sufficient learner centeredness. For the later, a mentoring system is a prerequisite. Research is unequivocal that mentoring supports and enhances reflective learning [21], [22], [23]. Regardless if reflection is part of the portfolio, a mentor is also considered to be essential in portfolio learning and the portfolio process [11], [16], [21], [24]. Given the previously mentioned lack of confidence or fear that the portfolio content is used ‘against’ the learner, it is important that the mentor is independent, and as noted by Dekker et al., not the daily supervisor or the head of the department [24]. 

In conclusion, the use of a portfolio, in combination with a mentor, helps and stimulates to look back and reflect at the collected information and this seems helpful for learning and further professional development. Pitfalls are time-constraints and an obligatory nature.

## The portfolio as an instrument for decision-making

Competency-based education needs continuous, elaborate and comprehensive feedback systems and assessment [25]. In post-graduate training, the use of a portfolio can facilitate the continuity and comprehensiveness of assessment by collecting information about the trainees progression towards outcomes, milestones, competencies and Entrustable Professional Activities (EPAs) [26]. 

For portfolio assessment as such, sufficient reliability and validity has been shown, given a number of qualitative criteria such as feedback cycles incorporated in the assessment process, and procedural safeguards in the assessment procedure [16], [27] , [28], [29]. Potential weaknesses are the time-consuming nature of assessment, need for an active feedback culture and acceptance by the users [30]. In addition, as expressed above, we need to be aware of the effects of the assessment of reflection. As expressed by Hodges et al., there is a tension between assessment and accountability on the one hand, and the requirement of trainees to reflect on their actions on the other hand [31], [32]. As indicated in the previous paragraph, reflection in medical education has its merits, it is essential for learning from clinical practice and experiences, and therefore it should be common practice of every medical professional [33]. However, a careful balance should be sought, this could be a portfolio containing reflection in a dialogue with a mentor [33], with an assessment that is fit for purpose, meaningful and not judgmental. This format of assessment of reflection is certainly not easy or common within the field of medical education.

Nevertheless, a portfolio can be a useful and meaningful instrument for the assessment of competencies if certain principles of continuity, comprehensiveness and opportunities for a meaningful dialogue on feedback are applied in the design of the assessment process. Such a design of continuous assessment was advocated by van der Vleuten and Schuwirth [34], [35]. In their model of programmatic assessment, assessment activities are rich in feedback and informative, they are purposefully chosen, aggregated and arranged in time. This will provide a longitudinal flow of information about the trainee, which the trainee can use to learn from feedback and assessment and to plan future learning opportunities. In this model, there are no single (high-stake) decisions, rather the aggregation of all information available is used to come to pass-fail or high-stake decisions, or a decision on promotion. This model resonates with the view of Eva et al., that a continuous model of assessment has the potential to mitigate potentially unwanted features of competency-based assessment. A program of assessment could help to overcome the psychometric challenges and context-specificity of competency assessment, and combine the formative and summative assessment, by broad and purposeful sampling [36]. In addition, in competency-based education, progression through residency is no longer simply time-dependent. Trainees can progress and complete residency at the time that final outcomes or qualifications are met. Therefore, decisions on the level of expected proficiency need to be made. Ten Cate introduced the Entrustable Professional Activity (EPA) to facilitate and aid in the decision on proficiency level [37]. An EPA is a professional unit or task that is entrusted to the trainee to perform without direct supervision once the trainee has shown or provided evidence that the expected level of competency has been reached (37). The Entrustment decision itself resembles the assessment of a portfolio in that multiple sources of information are used, by different supervisors, in various context, to support the validity of the decision [38]. The models and theoretical foundation of entrustment decisions are complex and currently under deliberation, e.g. by the International Competency-Based Medical Education Collaborators (38). It should be kept in mind that an EPA represent a complex set of behaviors that have to be recognized and observed by clinical supervisors in the workplace. Oerlemans et al. suggested that observing clinical performance during a series of encounters by the same supervisor revealed consistent behavior and added valuable information on the trainees’ performance [39]. This aligns with the broad and purposeful sampling strategy used in programmatic assessment. In addition, clinical supervisors need to be supported and trained, Calaman et al. described the development of standard-setting videos in the context of EPAs and entrustment decisions to support direct observation of trainee performance by clinical supervisors [40]. An electronic portfolio format could support the entrustment decision process by integration of information through automatically generated overviews of forms, narrative feedback and levels of proficiency. In the e-portfolio, a timeline per EPA can be presented or generated in which the decisions and levels are shown and the supervisor can easily navigate back to the underlying forms and information.

In conclusion, the portfolio as an instrument for continuous, programmatic assessment is usable in post-graduate medical training, as assessment instruments (such as mini-CEX, observational instruments, technical skills assessments), and feedback instruments (multi-source feedback) are usually operational and collected in the resident portfolio. EPAs are increasingly used in post-graduate education. The theoretical foundation of entrustment decisions is complex. An electronic portfolio format could be helpful in supporting the process and procedures of entrustment decisions. 

## The portfolio as a quality assurance tool

A portfolio can also be used as a quality assurance instrument, e.g. how many procedures have been performed, success rate of procedures, registration of professional development or courses, patient evaluations etc. The purpose of the portfolio will then be more tailored to a dossier function, in which information is being collected and if needed aggregated and reported. The portfolio as a dossier for management issues can be applicable to both individual trainees and (senior) physicians, as for training programs. An electronic format has greatly enhanced the potential of portfolios as a quality assurance tool as data can now easily be aggregated and compared. In undergraduate programs, the portfolio has also been used to get insights in the hidden curriculum, e.g. to monitor for the practices and values that are taught or transmitted through role modeling [41] or to explore the content of e-portfolio on gender issues entries, a topic often taught in the hidden curriculum [42]. In post-graduate education, the portfolio could also fulfil this role on the quality of clinical teaching, e.g.by monitoring the richness and quality of the narrative feedback.

In addition, many countries have reformed the official requirements for revalidation processes for physicians [http://www.gmc-uk.org/guidance/good_medical_practice.asp], [43], [44], [45], in which evidence of (reflective) activities and practice are often mandatory elements. For these revalidation procedures, portfolios are used [46]. Given the current commonality of portfolio use in post-graduate medical training, this will most likely be a natural continuation of portfolio use once residency training has ended. For senior doctors, this may be the first time that they have to complete a portfolio. As already addressed, the bureaucracy and often complexity of portfolio or in this case revalidation procedures have the danger of transgressing into a requirement, and the process is perceived as less meaningful for stakeholders. 

## Three purposes: one portfolio for each or all in one portfolio?

We have discussed three purposes of portfolios, i.e. to enable reflection, to assess competencies and as an instrument for quality assurance processes. As was already evident, there is a natural overlap in these purposes, one cannot reflect if no information is being collected, and information on performance or proficiency level is used for assessment and entrustment decisions. A potential pitfall of having everything in one portfolio is that it becomes too complex, bureaucratic and time-consuming. The challenge is to keep portfolio processes meaningful for all stakeholders. We argue that a portfolio can serve all three purposes, but much is dependent on the implementation and how it is used. The different goals of the portfolio should be clear for all stakeholders, residents, clinical teachers and supervisors and mentors. Instruction and communication should be transparent and clearly designed. The mentor is an important source of information for the learner and should serve as an intermediary between the learner/ resident and the program. We believe that a mentor also has an important role in the above mentioned problems with acceptance or reluctance to work and use the portfolio for learning. Best-practice examples for implementation of portfolio in undergraduate and post-graduate medical programs can be found in Driessen et al. [47], Dannefer et al. [48], and Fung et al. [49].

In conclusion, the portfolio needs to be a learner chart [33], in which information is collected (dossier), used for learning guided by a mentor or coach (reflect) and serves as the basis for decisions on proficiency or outcome requirements (assess). Then it can be a learner chart that comprehensively documents the trainees’ progress and which is discussed between a trainee and a mentor to support the trainees’ development. When the portfolio is integrated in a program of assessment, and decision-making is removed from individual assessments and decisions are only based on longitudinally gathered assessment and learning information: then the portfolio becomes the key instrument that fully services learning, assessment and quality assurance.

## Competing interests

The authors declare that they have no competing interests. 
